# PET/CT Imaging of c-Myc Transgenic Mice Identifies the Genotoxic N-Nitroso-Diethylamine as Carcinogen in a Short-Term Cancer Bioassay

**DOI:** 10.1371/journal.pone.0030432

**Published:** 2012-02-02

**Authors:** Katja Hueper, Mahmoud Elalfy, Florian Laenger, Roman Halter, Thomas Rodt, Michael Galanski, Juergen Borlak

**Affiliations:** 1 Institute for Diagnostic and Interventional Radiology, Hannover Medical School, Hannover, Germany; 2 Department of Molecular Medicine and Medical Biotechnology, Fraunhofer Institute of Toxicology and Experimental Medicine, Hannover, Germany; 3 Institute for Pathology, Hannover Medical School, Hannover, Germany; 4 Centre for Pharmacology and Toxicology, Hannover Medical School, Hannover, Germany; National Institute of Health, United States of America

## Abstract

**Background:**

More than 100,000 chemicals are in use but have not been tested for their safety. To overcome limitations in the cancer bioassay several alternative testing strategies are explored. The inability to monitor non-invasively onset and progression of disease limits, however, the value of current testing strategies. Here, we report the application of *in vivo* imaging to a c-Myc transgenic mouse model of liver cancer for the development of a short-term cancer bioassay.

**Methodology/Principal Findings:**

μCT and ^18^F-FDG μPET were used to detect and quantify tumor lesions after treatment with the genotoxic carcinogen NDEA, the tumor promoting agent BHT or the hepatotoxin paracetamol. Tumor growth was investigated between the ages of 4 to 8.5 months and contrast-enhanced μCT imaging detected liver lesions as well as metastatic spread with high sensitivity and accuracy as confirmed by histopathology. Significant differences in the onset of tumor growth, tumor load and glucose metabolism were observed when the NDEA treatment group was compared with any of the other treatment groups. NDEA treatment of c-Myc transgenic mice significantly accelerated tumor growth and caused metastatic spread of HCC in to lung but this treatment also induced primary lung cancer growth. In contrast, BHT and paracetamol did not promote hepatocarcinogenesis.

**Conclusions/Significance:**

The present study evidences the accuracy of *in vivo* imaging in defining tumor growth, tumor load, lesion number and metastatic spread. Consequently, the application of *in vivo* imaging techniques to transgenic animal models may possibly enable short-term cancer bioassays to significantly improve hazard identification and follow-up examinations of different organs by non-invasive methods.

## Introduction

Hepatocellular carcinoma (HCC) is frequently observed in cancer bioassays as a result of lifetime exposure to either experimental drugs or a diverse range of chemicals. Specifically, carcinogens can be distinguished by their mode of action some of which exert activity either through DNA damage and thus are defined as genotoxic carcinogens such as N-nitrosodiethylamine (NDEA), while others do not harm DNA but cause perturbations in biological process that ultimately leads to uncontrolled growth and are termed non-genotoxic carcinogens. This latter group of carcinogens is inherently more difficult to predict. Furthermore, drugs and chemicals may not be by themselves tumorigenic but promote tumor growth.

Furthermore, while the process of evaluating the safety of chemicals is internationally harmonized, it remains costly and time consuming. In order to reduce the time needed for an evaluation of the carcinogenic risk of drugs and chemicals, novel transgenic animal models have been developed and biological read outs from such models are compared to the established cancer bioassay. However, the common cancer bioassays require an observation period of 2 years and utilize a large number of animals that are subsequently subjected to histopathology examinations [1].

To overcome limitations in the cancer bioassay several alternative testing strategies are being explored, either involving genetically modified animals, *in vitro* cell based assays or computerized models [2]–[7]. In this regard genetically modified laboratory animals may prove valuable in the early detection of liver carcinogens and thus may enable short-term carcinogenicity studies. So far, several knock-out models as well as transgenic mouse models of hepatocellular carcinoma have been reported [8]. For example the response to the genotoxic carcinogen NDEA was evaluated in a p53-deficient [9] as well as in the rasH2 transgenic model [10]–[12]. Unfortunately, both models failed to identify NDEA as a liver carcinogen in short-term cancer bioassays rendering this strategy less robust for the detection of organ specific carcinogens with these two genetic models.

Additionally, the standard rodent carcinogenicity assays is unable to monitor non-invasively onset and progression of disease. Hence, imaging techniques like micro computed tomography (μCT) and micro positron emission tomography (μPET) may become a method of choice in preclinical studies to detect *in vivo* tumor lesions and to quantify tumor load. *In vivo* imaging will thus contribute to the refinement of cancer bioassays and offer additional information, such as an identification of metastatic spread and secondary tumor growth in a timely fashion [13].

Indeed, the recent advancement in small animal imaging technologies encouraged us to examine the utility of μCT and μPET for the detection of tumor lesions in a transgenic mouse model of liver cancer. With such enabling technologies μCT delivers a resolution of anatomical structures of about 50 µm, therefore providing considerable morphological information. This requires, however, the use of an organ specific contrast agent to allow imaging of the parenchymatous morphology. Notably, the c-Myc transgenic mouse model efficiently develops liver cancer in a short period of time. In this genetic model, c-Myc is targeted to the liver by use of the α1-antitrypsine promoter which is exclusively activated in the liver. This model was originally developed by Dalemans et al. [14], but has not been explored for its usefulness in short-term cancer bioassays, as yet. Notably, human molecular pathology studies identified c-Myc as hyperactive and overexpressed in the majority of human hepatocellular carcinoma [15] therefore providing a rational for an evaluation of this disease model.

While a few reports describe the application of anatomical and metabolic imaging of primary liver malignancies in rodents [16]–[19], the aim of this study was firstly, to develop strategies and define parameters for the detection and the quantification of liver lesions by *in vivo* contrast-enhanced μCT and ^18^F-FDG μPET metabolic imaging of glucose uptake and secondly, to evaluate the accuracy of these methods when compared with histopathology and thirdly, to determine the utility of the c-Myc transgenic mouse model in accurately predicting the liver carcinogen NDEA in short-term bioassay and thus to evaluate whether a combination of *in vivo* imaging modalities and the use of genetic mouse models may help to develop short-term cancer bioassays for the early detection of hazardous drugs and chemicals.

## Materials and Methods

### Transgenic tumor model

All animal work followed strictly the Public Health Service Policy on the humane care and use of laboratory animals. Permission to carry out the study was obtained by the animal welfare ethics committee of the city of Hannover, Germany (Tierversuchsvorhaben 33.9-42502-04-08/1619).

c-Myc transgenic mice were the kind gift of Dalemans et al. [14]. The animals were maintained as homozygotes in the C57/Bl6 background and this background is widely used in transgenic disease models such as the rasH2 and p53 deficient model. Notably the transgene (see Figure S1) consists of the c-Myc open reading frame and regulatory sequences of the α1-antitrypsine promoter to allow liver specific gene expression of c-Myc. This genetic disease model has an incidence of liver cancer of 100%.

The transgene was detected by PCR using the forward primer: 5′-CACTGCGAGGGGTTCTGGAGAGGC-3′ and the reverse primer: 5′-ATCGTCGTGGCTGTCTGCTGG-3′. The following PCR assay conditions were used: 15 min, 95°C, 1 min 60°C, 1 min 70°C, 1 min 95°C, 31 cycles.

Altogether 120 c-Myc transgenic mice were examined by *in vivo* imaging and/or histopathology, while the non-transgenic controls were studied by histopathology only, as these animals do not have tumors. Mice were kept as groups of animals with 1 to 4 mice per cage on sawdust in a 12 hour light-dark cycle, and 50% relative humidity and an ambient temperature of 22°C. The animals received standardized chow and drinking water ad libitum (Zucht, ssniff M-/, 10 mm, complete diet for mice, ssniff Specifications GmbH, DE-59494, www.ssniff.de).

### Study design and treatment of animals with NDEA, Butylated Hydroxy Toluene (BHT) and paracetamol

The animals were divided into 7 groups of 24 animals each and consisted of n = 12 males and n = 12 females. Treatment of transgenic mice with intraperitoneal injection of NDEA, BHT or paracetemol (Sigma Aldrich, Germany with a purity of >99%), was started at the age of 2 months. The transgenic and the non-transgenic control animals were also treated with the vehicle only, i.e. corn oil or physiological saline (Sigma Aldrich, Germany). NDEA mice were treated once a week by injection of 75 µg/g NDEA in saline over a period of 6 weeks while animals receiving 300 µg/g BHT in corn oil were treated once a week for 8 weeks. Moreover, 100 µg/g paracetamol in saline served as a non-carcinogenic hepatotoxin, and this drug was given once daily for 5 days per week over a period of 8 weeks (see Table 1 for the dosing regime).

**Table 1:** Study groups and imaging protocol.

	Animals	Treatment (vehicle)		Dose (duration)	Age at which CT/PET examination and histopathology was done			
1	transgenic	NDEA	(0.9% NaCl)	75 µg/g (once weekly for 6w)	4 m	5.5 m	7 m	**8.5 m**
2	transgenic	BHT	(corn oil)	300 µg/g (once weekly for 8w)	4 m	5.5 m	7 m	**8.5 m**
3	transgenic	paracetamol	(0.9% NaCl)	100 µg/g (5 days per week for 8w)	4 m	5.5 m	7 m	**8.5 m** *
4	transgenic	-	(0.9% NaCl)	(once weekly for 6w)	4 m	5.5 m	7 m	**8.5 m** **
5	transgenic	-	(corn oil)	(once weekly for 8w)	4 m	5.5 m	7 m	**8.5 m** **
6	non-transgenic	-	(0.9% NaCl)	(once weekly for 6w)	4 m	5.5 m	7 m	**8.5 m** *
7	non-transgenic	-	(corn oil)	(once weekly for 8w)	4 m	5.5 m	7 m	**8.5 m** *

Histopathology was carried out for all groups and for all time points (4, 5.5, 7, 8.5 months).

*No CT/PET imaging was done,

**CT/PET was acquired at the age of 8.5 months only. m, months; w, weeks.

As C57Bl/6 mice are resistant to hepatocarcinogenesis induced by NDEA [9] a non-transgenic NDEA and BHT treatment group was not included in the study design It is of considerable interest that a similar lack of sensitivity was reported for p53 deficient and for rasH2/CB6F1 transgenic mice which were bred in the same C57BL/6 background [11]. Likewise, it was shown earlier that BHT enhances tumor formation only if administered after exposure to the genotoxic agent urethane, but has no effect on its one [20] or is even protective, if given before a carcinogen [21], [22]. A summary of the treatment schedule is given in Table 1.

### Dose selection

Based on previously published data, where different doses of NDEA were investigated (dose range 75 to 200 µg/g) to cause tumor formation in non-transgenic rodents [23], [24], [25], [26], a dose of 75 µg/g body weight was selected and given once weekly for 6 weeks by intraperitoneal administration. In the case of BHT a dose of 300 µg/g bodyweight was administered. This dose was reported to cause tumor induction in the liver [20]. Finally, paracetamol served as the non-carcinogenic hepatotoxin and a dose of 100 µg/g bodyweight was given i.p. once daily for 5 days per week over a period of 8 weeks

### In vivo imaging of transgenic animals by μCT and μPET

At four different time points – i.e. at the age of 4, 5.5, 7 and 8.5 months - *in vivo* μCT and μPET imaging were employed; animals were sacrificed afterwards for histopathology.

All imaging procedures were performed under inhalation anesthesia with isoflurane (Isoba vet., Essex Pharma, Germany) at a concentration of 4% for induction of anesthesia and 1–2% for maintenance. Mice were placed in prone position on a temperature controlled bed at 39°C (T/Pump, Gaymar, Orchard Park, NY, USA) allowing changes between imaging modalities without repositioning and isoflurane was supplied via a nose cone (Summit Anesthesia Solutions, Bend, OR, USA). The animal respiration was spontaneous, and the breathing was monitored continuously using a small pressure transducer (Biovet, m2m imaging, Newark, NJ, USA). Breathing was maintained at a rate between 60 and 100 per minute. After image data acquisition the recovery time of the animals from anaesthesia was usually less than five minutes. Overall the procedures were well tolerated.

Specifically, sequential μCT and ^18^F-FDG μPET imaging were carried out with a total of 60 animals as shown in Table 1. Mice were imaged at the age of 4 (1st sacrifice), 5.5 (2nd sacrifice), 7 (3rd sacrifice) and 8.5 months (4th sacrifice). The transgenic control animals were examined at the age of 8.5 months only (4th sacrifice), although some explorative contrast-enhanced imaging was carried out at the age of 2, 5 and 7 months. All animals were sacrificed 2 days after the PET imaging. At this time the radioactive tracer declined to below the level of detection. Imaging findings were corroborated by standard histopathology as detailed below.

### Contrast enhanced μCT imaging

All animals were fasted prior to imaging for about 6 hours. Three hours prior to CT scans anaesthetized mice were given an intravenous injection of a liver-specific iodinated contrast agent (DHOG, Fenestra LC, ART Inc., Saint-Laurent, Canada) at an approximately volume of 200–300 µl (10 µl/g bodyweight) into the tail vein. The image data acquisition was carried out as recommended by the manufacturer and previously published protocols [18], [19].

The μCT scan was done with a high-resolution small animal computed tomography scanner (eXplore Locus, GE Healthcare, Chalfont St. Giles, UK). The scan parameters were set as follow: tube voltage 80 kVp, tube current 450 µA, number of acquisition 360, number of views 720, exposure time 100 ms, one average per frame, axial field-of-view 33 mm. Scans were recorded without respiratory gating. Total scan duration was about 12 minutes.

Image data was reconstructed using a cone-beam algorithm on an 8-node linux cluster. The resulting voxel size of the isotropic dataset was 45 µm. Arbitrary attenuation values were converted to the Hounsfield scale using a calibration phantom with water, air and bone inserts.

### μPET imaging

Anaesthetized mice were given an intraperitoneal injection (i.p.) of 10 MBq (^18^F)-2-fluoro-2-deoxyglucose in a total volume of 50–100 µl sterile isotonic saline solution supplied by the Department of Nuclear Medicine, Hannover Medical School, Germany. Animals were subjected to sequential CT and PET imaging which necessitated multiple injections into the tail vein to possible cause vascular lesions and embolization at the injection site. As CT scans required i.v. administration of Fenestra (see above) ^18^F-FDG was given by i.p. injection. This may delay standard acquisition of FDG, however, did not influence the overall interpretation of data with glucose uptake being proportional to tumor growth as evidenced in the present study. Therefore, after CT image acquisitions ^18^F-FDG was administered to mice by i.p. injection. The animals remained anesthetized and after about 35 min were transferred on the same bed/position from the CT scanner to the PET scanner. Static images were acquired exactly after 45 minutes of injection of the tracer using a high-resolution small animal PET camera (eXplore Vista, GE Healthcare, Chalfont St. Giles, UK). Total acquisition time was 30 minutes for a single bed position. Images were corrected for random events and scatter prior to reconstruction with a 3D-FORE/2D-OSEM iterative algorithm. No attenuation correction was used.

### Image analysis

μCT datasets were visualized and analyzed using the software packages Microview 2.2 (GE Healthcare, Chalfont St. Diles, UK), MeVisLab 2.0 (MeVis Medical Solutions AG, Bremen, Germany) and OsiriX (v.3.7.1 32-bit, Pixmeo Sarl). Total liver volume was calculated using the LiveWireMacro module (MeVisLab 2.0) which utilizes a contour-based semi-automatic segmentation method. Focal liver lesions were counted and quantified by 2D-measurement of the largest diameter. The diameter (d) of the lesions was used to estimated the tumor volume by the following formula: tumor volume (V) = 1/6×π×d^3^. The volumes of all liver lesions were added to determine the total tumor volume. The tumor percentage of the liver was calculated as the ratio of the total tumor volume (ml) and the total liver volume (ml).

Rigid registration of PET and CT datasets was based on anatomical landmarks and used to generate fused datasets. Regions-of-interest (ROI) were manually defined for focal liver lesions of a diameter above 5 mm as detected in μCT and ^18^F-FDG μPET imaging. The background (non-tumor) signal was determined by placing a ROI in the tumor-free liver parenchyma and the maximum count per volume was determined for each ROI to estimate the tumor-to-non-tumor ratios.

### Histopathology

Two days after the last imaging mice were euthanized and liver and lung were removed for histopathology. The entire lungs and livers were immersed in buffered 4% formaldehyde and embedded in paraffin by standard laboratory procedures. Subsequently, 4–5 µm sections of the blocks spaced at an interval of 500 µm were prepared from the central core of the embedded tissue and stained with H&E. These slides were examined using an Olympus BX51 microscope at a 40× original magnification. 4 different morphological findings were recorded: 1) regular hepatic parenchyma as defined by cell size and preservation of architecture, 2) diffuse toxic injury of the liver parenchyma as defined by ballooning, degeneration, but preservation of architecture, 3) dysplastic nodules with nodular aggregates of enlarged liver cells with low grade cellular dysplasia, 4) hepatocellular carcinoma as defined by cellular and architectural atypia with multilayered trabecular or pseudoglands. Type, number and size were recorded for all lesions and compared with the results of the radiological imaging. Furthermore, the tumor load was scored semiquantitatively using 5% increments.

### Statistical analysis

The *in vivo* imaged liver volume and the *ex vivo* measured liver weight were recorded and the liver volume and the tumor volume were computed and defined as the tumor percentage. All values are given as mean±SEM. Statistical significance was determined by the Pearson correlation test (r is the correlation coefficient) and imaging findings were validated by histopathology as described above.

The tumor-to-non-tumor ratios were defined for small (<5 mm), medium (5–10 mm) and large lesions (>10 mm) and statistical significant differences were determined using the one-way ANOVA and the Bonferroni post-test.

At different time points and for all study groups the means and standard deviations of the tumor percentage, the tumor multiplicity and the tumor-to-non-tumor ratio were compared. The one-way ANOVA and the Bonferroni post test were used to evaluate statistical significance (p-value cutoff determined as 0.05).

All statistical analyses were performed and visualized using SPSS Statistics 17.0 and GraphPad Prism 5.0 (GraphPad Software, Inc.).

## Results

### Contrast-enhanced liver morphology and glucose metabolic imaging

**Figure 1:**
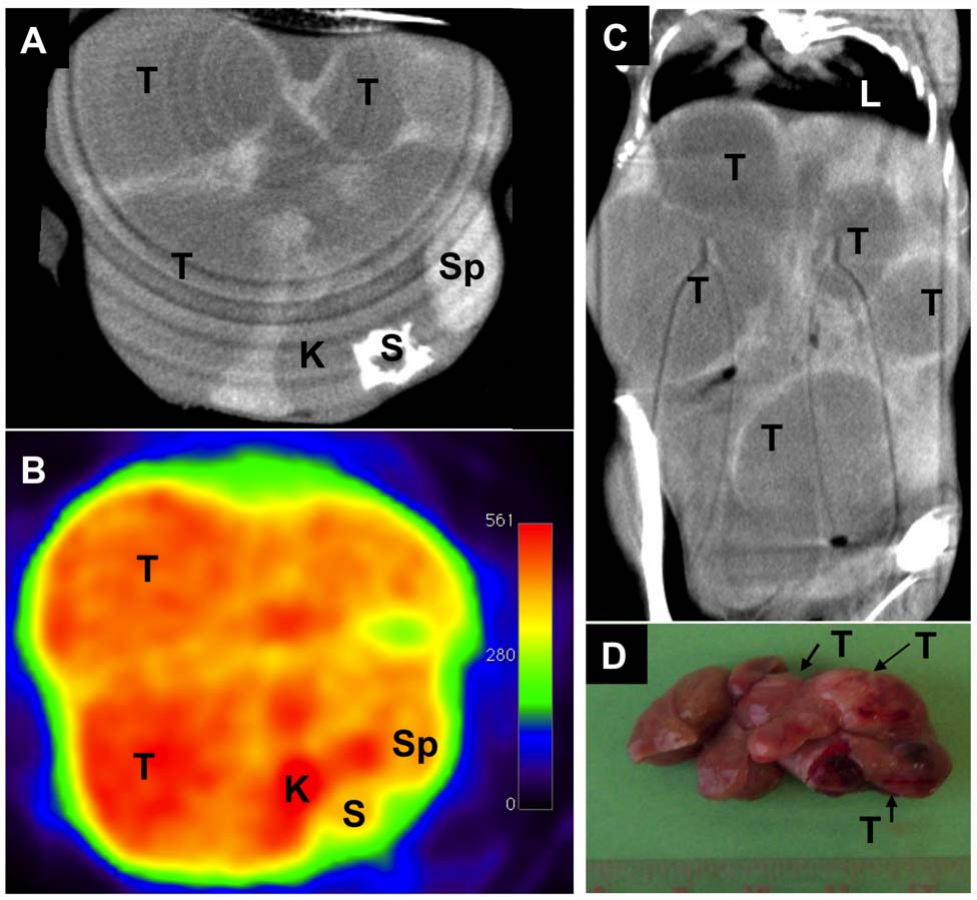
Contrast-enhanced CT and ^18^F-FDG-PET of the liver of an NDEA-treated c-Myc transgenic mouse. Axial slices of a CT (A) and an FDG-PET scan (B) as well as coronal slices of a CT scan (C) and macroscopic view (D) of an explanted liver of an NDEA treated mouse at the age of 7 months. When compared to the normal liver parenchyma tumors are hypodens at CT as the uptake of the liver specific contrast agent is reduced. PET scans reveal tumor lesions by an increased tracer uptake due to enhanced glucose metabolism. Because of the advanced stage of disease tumors of different size had merged together. K = kidney, L = lung, S = spine, Sp = spleen, T = tumor.

At the beginning of the study none of the transgenic animals had liver tumors or precursor lesions while at the end of the study a total of 244 liver lesions were detected that varied in size between 0.9 mm and 25.0 mm. Notably, intravenous injection of the liver-specific iodinated contrast agent DHOG defined tumor lesions as hypodense as compared to the normal surrounding liver parenchyma (Figure 1). Lesions of a size of >1 mm could be identified with certainty by μCT but not with FDG μPET imaging for lesions of <5 mm. In animals with a high tumor load individual lesions could not always be resolved due to individual tumors that had merged together (collision tumors).

### Assessment of growth and multiplicity of tumor lesions

**Figure 2:**
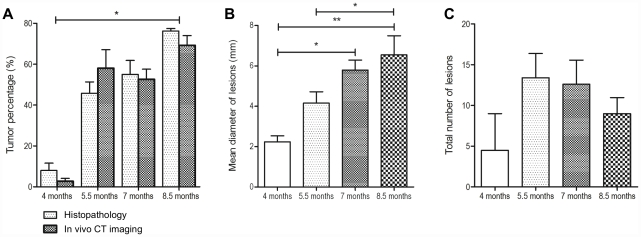
Time dependent changes in tumor growth of NDEA-treated c-Myc transgenic mice. (A) The organ and tumor volume was assessed by CT and by histopathology. Depicted is the percentage tumor volume at different stages measured either by histopathology (light gray) or by contrast enhanced CT imaging (dark gray). The mean diameter of lesions (B) and the total number of lesions (C) are shown. *p<0.05. **p<0.01.

Treatment of transgenic mice with the genotoxic hepatocarcinogen NDEA induced rapidly the development of HCC. At the age of 4 months (1st sacrifice) 1 out of 6 animals had HCC, and the tumor incidence was already 100% at the age of 5.5 months (2nd sacrifice). By CT imaging the liver and tumor volume was determined and the resultant ratio was defined as tumor percentage (Figure 2A). Overall, there was no difference in tumor incidence or tumor volume when male and female c-myc transgenic mice treated with NDEA were compared (Table 2).

**Table 2:** Mean tumor volume for NDEA treated c-Myc transgenic mice separated by gender.

	4 months		5.5 months		7 months		8.5 months	
Gender	CT	Histo	CT	Histo	CT	Histo*	CT	Histo
Male	0±0	2±2	47±30	45±19	46±24	48±17	68±13	80±0
Female	5±5	23±18	65±32	47±23	50±20	75±0	74±0	73±3

Data were obtained by CT and histopathology and are defined as mean percentage tumor volume and standard error of the mean for NDEA treated animals. Data are broken down by gender. At the age of 5.5 months the tumor incidence was 100% for male and female animals. No gender specific difference in tumor volume was observed except for histopathology findings at the age of 7 months;

*P<0.05.

The data was validated by histopathology as detailed below. There was good agreement between the histopathology and *in vivo* imaging results. With regard to tumor growth the greatest difference was observed between the 1st and 2nd sacrifice where CT-scans defined percentage tumor volumes of 2.8% and 58.0%, respectively. At the age of 8.5 months (4th sacrifice) the percentage tumor volume was 69.3%. The diameter and the numbers of lesions were determined by CT imaging and by histopathology. With NDEA the tumor lesions increased time dependently from 2.2±0.3 mm (1st sacrifice) to 6.5±0.9 mm (Figure 2B). Likewise, tumor multiplicity for NDEA treated animals increased from the first to 2nd sacrifice but decreased afterwards possibly as a result of merging tumors (Figure 2C).

**Figure 3:**
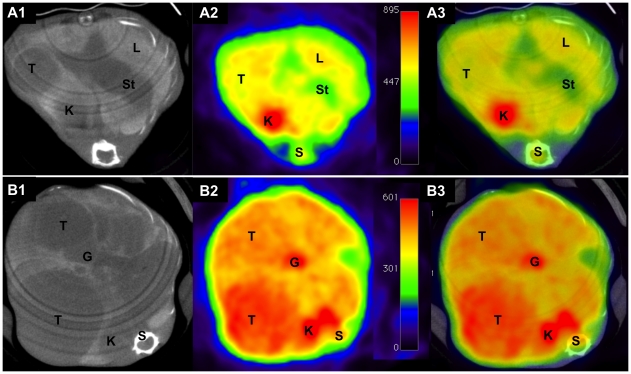
Contrast-enhanced CT, ^18^F-FDG-PET and fused images of c-Myc transgenic mice treated with NDEA at the age of 5.5 and 7 months. (A) CT image demonstrates a single tumor lesion (A1) in a 5.5 months old mouse without increased ^18^F-FDG uptake (A2). The fused CT and PET image is depicted in A3. (B) At the age of 7 months expansive tumor growth (B1) as well as an increased tracer uptake in hepatocellular carcinoma (B2) is observed. The fused CT and PET image is depicted in B3. G = gallbladder, K = kidney, L = liver, S = spine, St = stomach, T = tumor.

The time dependent tumor growth is also depicted in Figure 3, where CT and PET scans of NDEA treated animals were acquired at the age of 5.5 and 7 months, respectively. Here the glucose metabolism in liver lesions was determined by in vivo ^18^F-FDG μPET imaging.

To allow for accurate anatomical localization of focal ^18^F-FDG uptake, registration and fusion of μCT and μPET datasets were acquired prior to quantitative analysis of glucose imaging (see Figure 3). For this purpose the animals were placed in prone position on a multimodality temperature controlled bed without repositioning of animals when imaging modalities were changed. Thus, animals were kept under inhalation anesthesia and in the same bed position between CT and PET scan to allow registration of scans.

The ^18^F-FDG-uptake was mainly homogenous amongst the liver lesions. Some larger lesions displayed an inhomogeneous uptake of ^18^F-FDG that was particularly prominent in the peripheral parts of the tumor. Inhomogeneous tracer uptake was associated with cystic and necrotic changes of the tumor adjacent to vital tumor tissue as evidenced by histopathology.

**Figure 4:**
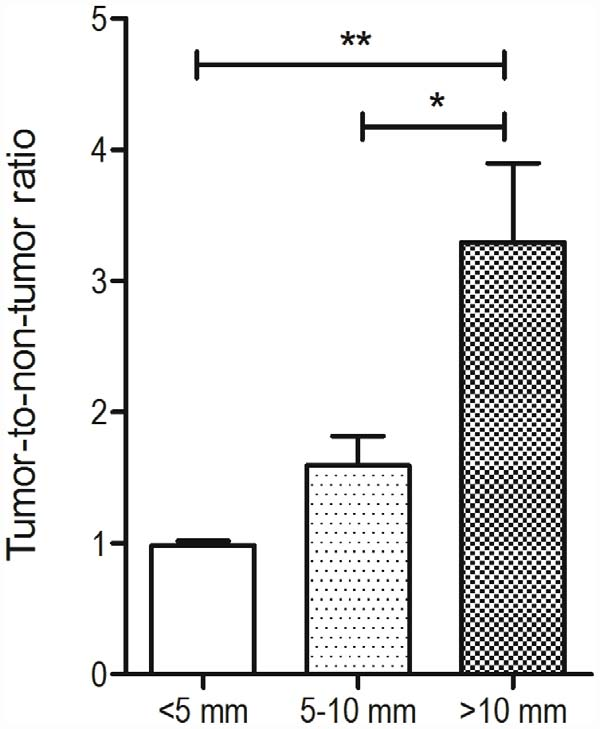
^18^F-FDG uptake to determine the tumor-to-non-tumor ratios in tumors of different size. Tumor-to-non-tumor ratio, determined by PET glucose imaging, was significantly increased in lesions >10 mm. *p<0.05. **p<0.01.

In general, the mean tumor-to-non-tumor ratio was dependent on the tumor size (Figure 4). For lesions larger than 10 mm the tumor-to-non-tumor ratio was 3.3±0.6 and determined to be statistically significantly increased (p<0.05) when compared to lesions with a diameter of 5 to 10 mm (1.6±0.2). For smaller lesions the tumor-to-non-tumor ratio was 0.98±0.03, therefore suggesting no increase in ^18^F-FDG-uptake when compared to the normal liver parenchyma.

**Figure 5:**
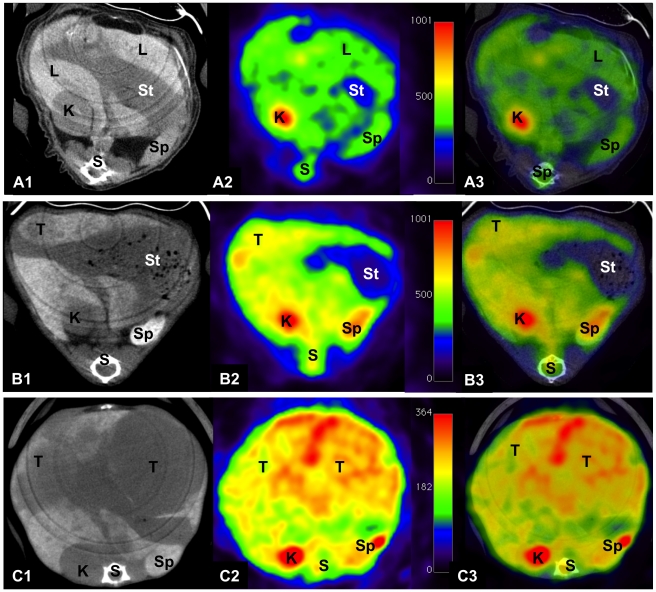
Fused μPET/μCT images of the liver of c-Myc transgenic mice treated with physiological saline, BHT or NDEA. Depicted are the liver morphology as determined by CT (A1, B1,C1), the glucose metabolism (A2, B2, C2) and fused PET and CT scans (A3, B3, C3) of transgenic animals treated with either physiological saline (A), with BHT (B) or with NDEA (C) at the age of 8.5 months. Note, after treatment with NDEA expansive tumor growth with large increase of liver weight and compression and displacement of adjacent organs was observed. Here, the lesions showed an increased 18F-FDG uptake. In contrast, in corresponding control animals treated with physiological saline no liver lesions were observed. After treatment with BHT small hypodens lesions are noticed, but PET did not show an increased 18F-FDG uptake. K = kidney, L = liver, S = spine, Sp = spleen, St = stomach, T = tumor.

Figure 5 summarizes PET/CT imaging and modality fusion of images obtained from transgenic mice treated with either saline (Figure 5A), BHT (Figure 5B) or NDEA (Figure 5C). Note, in 1 out of the 6 animals treated with BHT a tumor of >10 mm was observed.

Moreover, treatment of transgenic animals with the hepatotoxin paracetamol did not induce tumor growth as determined by histopathology (see below).

### Validation of imaging results by histopathology

**Figure 6:**
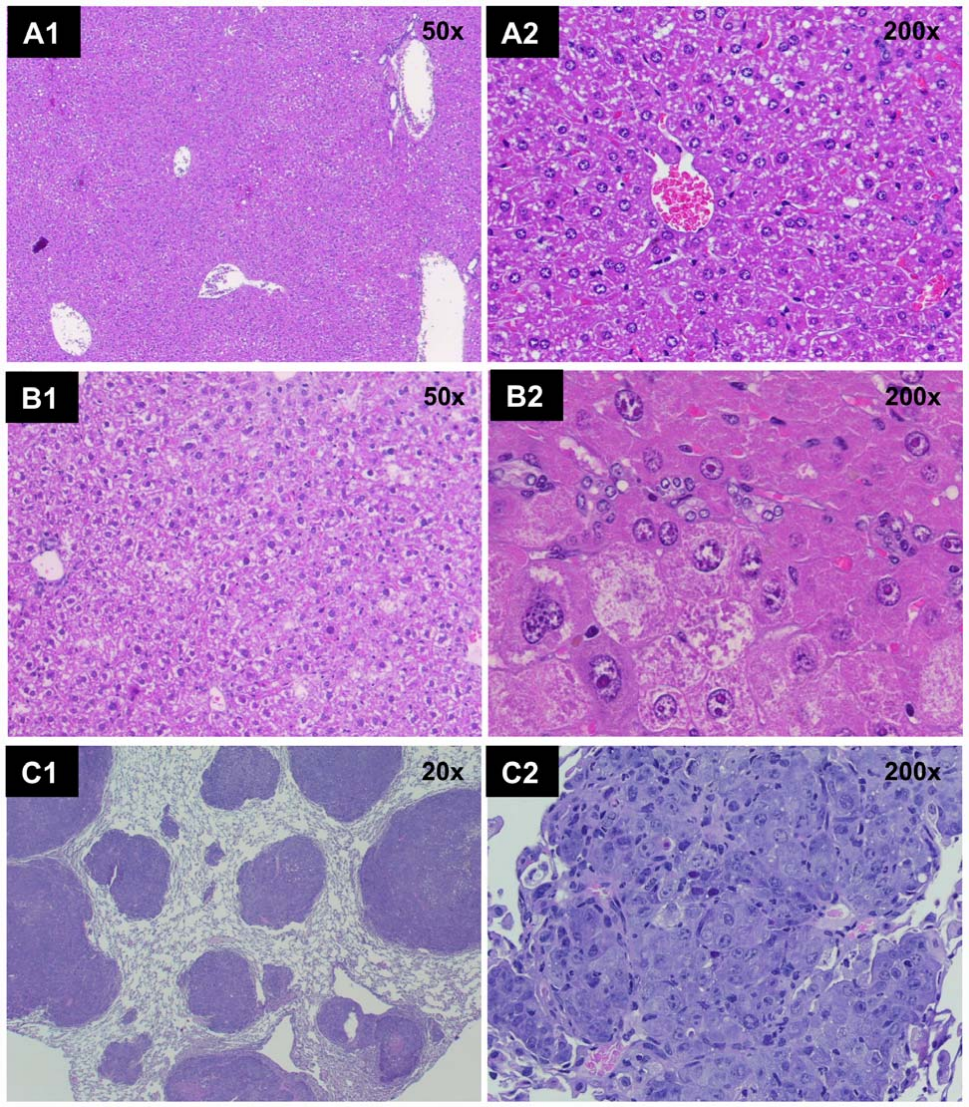
Histopathology of the liver of c-Myc treated transgenic animals. (A) Diffuse liver cell dysplasia of physiological saline ( = vehicle) treated transgenic mice at (A1) 50- and (A2) 200-fold magnification. (B) Large cell dysplasia of various degrees in BHT treated animals at (B1) 50- and (B2) 200-fold magnification. (C) Hepatocellular carcinoma of a transgenic mouse treated with the genotoxic carcinogen NDEA at (C1) 50 and (C2) 200-fold magnification.

Histopathology evidenced normal liver with complete preservation of lobular architecture, bile ducts and vasculature parenchyma in non-transgenic mice. In vehicle treated c-Myc transgenic animals diffuse dysplasia was observed (Figure 6A). A small number of transgenic animals receiving either physiological saline (1/24 animals) or corn oil (4/24 animals) as well as the BHT treated animals (3/24 animals) displayed uni- or multifocal dysplastic liver nodules replacing 10–80% of the liver parenchyma that ranged in size between 1 and 10 mm. These foci consisted of enlarged hepatocytes with a preserved nuclear cytoplasmic ratio, uni- to bicellular layers and an overall nodular architecture. Transgenic animals treated with paracetamol were similar in histopathology as observed with the vehicle treated controls (image not shown). One animal each of the corn oil and physiological saline treated transgenic animals displayed small foci of hepatocellular carcinoma. Here the cell size was smaller as compared to the dysplastic foci with an increase in nuclear size and a significant distortion of the architecture revealing multilayered trabecula and areas of cystic pseudoglands.

The number, size and biological aggressiveness of liver lesions were entirely linked to the NDEA treatment (Figure 6C). Hepatocellular carcinoma with multilayered trabecular architecture was observed as were pseudoglandular areas and sometimes cystic spaces within tumors. Of the 18 animals examined by histopathology 13 revealed hepatocellular carcinomas some of which (n = 10 animals) displayed dysplastic nodules adjacent to the hepatocellular carcinoma as well. Furthermore, 3 animals were identified with dysplastic nodules only while in 8 cases the HCC was also accompanied by marked bile duct proliferation. All HCC had areas of multilayered trabecular architecture, the majority also revealed pseudoglandular areas sometimes with large cystic and peliosis-like spaces. Due to the close spatial and temporal relationship of dysplastic nodules and HCC in the livers of the transgenic animals it seemed reasonable to define dysplastic nodules as precursors and early lesions of liver cancer. Here, the size of lesions may be used as a means to differentiate the liver lesions whereby dysplastic nodules were smaller than 5 mm and HCC were larger than 10 mm.

**Figure 7:**
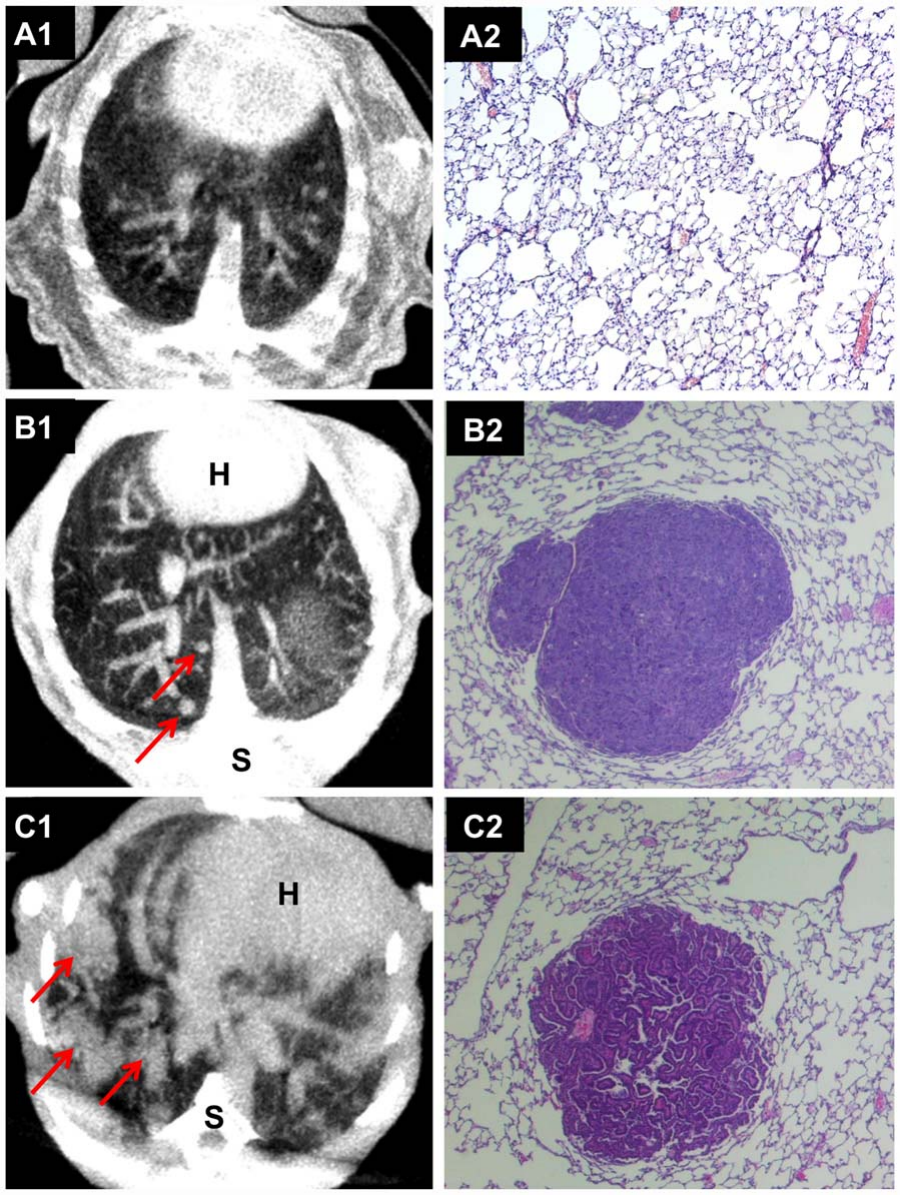
CT and histopathology of the lung of NDEA treated c-Myc transgenic mice. (A) Normal lung parenchyma of a vehicle treated control animal as shown by CT (A1) and by histopathology (A2). CT of NDEA treated animals at the age of 8.5 months. Depicted are lung nodules of different size (red arrows). Histopathology evidenced those lung nodules as metastasis of a poorly differentiated HCC (B2) and as well adenocarcinoma of the lung (C2).

Some animals treated with NDEA displayed metastasis of the primary liver cancer into lung (Figure 7B) but treatment with NDEA also induced primary lung cancer as evidenced by *in vivo* imaging and histopathology (Figure 7C). Thus, some animals were burdened by primary and secondary tumor growth in two different organs.

### Accuracy *of in vivo* imaging

There was a highly significant correlation between the liver volume, calculated by semiautomatic segmentation of CT-scans, and the liver weight, determined *ex vivo* (correlation coefficient r = 0.99, p<0.001). Additionally the calculated liver volume correlated with the estimated tumor volume (r = 0.94, p<0.001) (see Figure S2).

In Figure S3 liver to body weight ratio of transgenic mice treated with either NDEA or BHT is given. When the tumor volume determined by *in vivo* μCT and μPET imaging was compared with the tumor area defined by histopathology, a reasonable correlation was obtained (r = 0.82), therefore demonstrating the goodness of *in vivo* imaging (data not shown). Overall, all animals, defined by histopathology as positive for hepatocellular carcinoma, were accurately identified by *in vivo* imaging.

## Discussion

More informative and cost-effective testing strategies are in need to ensure protection of human health and the environment from risks that can be posed by chemicals. So far, the two-year rodent cancer bioassay is employed for hazard identification and risk assessment but prediction of carcinogenicity represents a problem which, as yet, could not be resolved satisfactorily [27], [28]. Much hope has been attached to the use of transgenic animal models but several models tested so far failed to meet expectations [9], [11], [29].

Here, we report findings with a c-Myc transgenic mouse models that accurately predicts risk for hepatocellular carcinoma for the genotoxic carcinogen NDEA. This transgenic model was evaluated in detail by histopathology and subjected to advanced *in vivo* imaging techniques. Indeed, contrast-enhanced μCT and ^18^F-FDG μPET metabolic imaging are facile methods for the detection of liver lesions and can be considered as robust *in vivo* imaging techniques to quantify lesions and to characterize tumor morphology [18], [19], [30]. In the present study, liver and tumor volume were quantified based on semiautomatic segmentation of CT scans [17]. A good correlation between tumor volume measured in CT and tumor load determined by histopathology was obtained. All animals, that displayed hepatocellular carcinoma, were accurately detected by CT and lesions as small as 1 mm in size could be defined. A notable finding of the present study is the ability to distinguish between dysplastic foci and tumor lesions by size, i.e. lesions <5 mm typically represented dysplastic nodules of different grade whereas hypodens lesions of >10 mm were hepatocellular carcinomas. In order to detected small liver lesions in CT, optimal contrast of the liver parenchyma is of crucial importance [13], [19]. This may not always be achieved. In the present study 10 out of 60 animals failed to present sufficient contrast and similar findings have been reported by others [31], [32]. Furthermore, high costs of the liver-specific contrast agent still limit its wide use in preclinical studies. In this regard PET imaging of ^18^F-FDG can be easily achieved and the radio-labeled glucose can be injected intraperitoneally. Specifically, ^18^F-FDG-PET provided additional information about tumor growth and differentiation as well as tumor necrosis and/or other cavities where no uptake of glucose is possible due to cysts within the tumor. Glucose uptake in tumors of ≥5 mm was statistically significantly changed when compared with tumors of >10 mm. Thus, for tumors of this size spillover and partial volume effects are negligible and this agrees well with previous findings [18]. However, an estimate of tumor-to-non tumor ratio in tumors of <5 mm can be confounded by spillover and partial volume effects, which especially occur in very small lesions and therefore the data needs to be viewed with caution and corrected. Overall, 18F-FDG uptake was highly dependent on the tumor size. Nonetheless, a definitive differentiation between HCC and precursor lesions such as foci of large cell dysplasia was not always possible, because some smaller HCCs did not show increased FDG-uptake when compared to the normal surrounding liver parenchyma (see Figure 4). Moreover, it should be noted that FDG-uptake reflects glucose metabolism, which is not necessarily specific for tumor infiltration. Indeed, increased FDG-uptake has also been reported for inflammation.

Despite this limitation a further advantage of *in vivo* imaging is the early detection of metastatic spread as well as growth of independent secondary carcinoma as observed in the present study whereby NDEA treated transgenic animals presented metastasizing liver cancer into lung but also displayed primary lung cancer growth; note, other investigators reported similar metastatic spread in a c-Myc and TGFα co-expressing transgenic mouse model [11], [33]. Thus, *in vivo* imaging offers the possibility to examine different tissues and organs within the same data acquisition.

The present study evidences the utility of μPET/μCT for quantitative evaluation of hepatocellular carcinoma. Follow-up examinations by serial and or sequential PET/CT scanning of the same animal provided additional information about tumor growth and development as well as metastatic spread and secondary tumor growth. This genetic tumor model may thus become an interesting preclinical testing strategy for liver carcinogens in an evaluation of experimental drugs and chemicals as part of short-term bioassays but studies with additional chemicals are needed to affirm the validity of the model.

As a proof of concept the genotoxic carcinogen NDEA, the weak tumor promoting agent BHT and the non-carcinogenic hepatotoxin paracetamol were examined. While the c-Myc transgenic model is genetically forced to develop hepatocellular carcinoma it can be used to assay activity of prototypic chemicals. It is of considerable importance that NDEA treatment of other genetic models, such as the rasH2 and p53 deficient mice failed to induce tumours within such a short period of time, i.e. 5.5 months [34], [35].

In the present study BHT, at the dose given, failed as a tumor promoting agent and for this non-genotoxic tumour promoting agent a threshold has been established. While some reports suggest BHT to promote liver cancer [36]–[38] a significant strain-dependent sensitivity for this chemical had been observed [39]. Thus, several strains of mice failed to respond to the effects of BHT even at high doses [36], [40], [41]. Possible BHT accelerates tumour growth in the c-Myc transgenic model at later time points or at higher doses. In the present investigation, however, histopathology defined mainly dysplastic nodules for this study group even at the age of 8.5 months.

In contrast, NDEA was effective in promoting carcinogenesis and treatment with this chemical caused metastasis of primary HCC into the lung as well as primary lung cancer growth therefore demonstrating high plasticity of the c-Myc transgenic model in some animals as early as the age of 5.5 months.

The effects of vehicle treatment were studied as well. None but one animal aged 8.5 months of the saline transgenic controls had tumors. A similar result was obtained when the mean diameter of lesions was compared between the NDEA and the corn oil as well as saline controls, i.e. approximately 9 mm for NDEA versus 3 and 2 mm for the corn oil and saline controls, respectively.

Because of its poor solubility in physiological saline NDEA was dissolved in corn oil. Note, the corn oil vehicle moderately affected tumor growth in transgenic animals but histopathology defined these CT findings primarily as foci of dysplastic nodules of different grade. Furthermore, all mice treated with corn oil showed fat accumulation in abdomen that may play role in tumor growth or induction of inflammation. Note, in the technical report 426 of the National Toxicology Program of the US corn oil was shown to cause hyperplasia and adenoma of exocrine organs. Thus, corn oil should be viewed with caution but treatment of the non-transgenic controls with the vehicle helped to better define the effects elicited by corn oil.

Finally, paracetamol treatment did not influence tumor growth and thus served as a hepatotoxic but non-carcinogenic control.

Overall, the c-Myc transgenic mouse model is responsive to the genotoxic carcinogen NDEA and may possibly differentiate between safe and hazardous chemicals as evidenced by the development of hepatocellular carcinoma already at the age of 5.5 months. The findings of the present study are also consistent with observations obtained with NDEA treated c-met knock-out mice where tumor growth was likewise accelerated [25]. Shortening the time of cancer bioassays will advance carcinogenicity testing but further studies with several agents are needed.

In conclusion, the combination of μCT and μPET imaging allowed *in vivo* detection and quantification of hepatocellular carcinoma. This proof of concept study provides evidence for the utility of the c-Myc transgenic model in a short-term cancer bioassay with the genotoxic agent NDEA. Validation of short term cancer bioassay is a priority task and will require the study of diverse chemicals with different mechanism of carcinogenicity. Nonetheless, the application of *in vivo* imaging techniques will significantly reduce the use of laboratory animals and likely enable earlier detection of hazardous drugs and chemicals.

## Supporting Information

Figure S1 **Scheme of the gene construct for the production of c-Myc transgenic mice.** (A) The gene construct contains the sequence of the α1-antitrypsine promoter thereby enabling liver specific gene expression of c-Myc. Based on ongoing research evidence was obtained that this genetic model of liver cancer undergoes different stages of disease with initial low and high grade dysplasia followed by different grades of hepatocellular carcinoma as evidenced by histopathology. (B) Depicted is an ethidium bromide stained agarose gel of a 303 bp PCR amplification product of the transgene detected in individual animals; the lanes 2, 3, 7, 10, 13, 15, and 16 refer to non-transgenic animals (no band) while the lanes 4, 6, 8, 9, 11, 12, 14, 17, 18 and 19 are transgenic animals. Note, lane 20 is a mock (water) negative control. MW = molecular weight marker.
(TIF)

Figure S2 **Correlation between liver weight, liver volume and tumor volume of c-Myc transgenic mice.** (A) demonstrates the close correlation between the liver weight and the liver volume (p<0.001, r = 0.99). (B) The correlation between liver volume and tumor volume – as determined by CT – is shown (p<0.001, r = 0.94).
(TIF)

Figure S3 **Liver volume in BHT- and in NDEA-treated c-Myc transgenic mice at different stages of disease.** Liver volume as determined by semiautomatic segmentation in contrast-enhanced computed tomography was constant over time in BHT-treated animals (A), whereas in NDE-treated animals it increased till the age of 8.5 months (B).
(TIF)
