# Supplementary figures and images for: PET/CT Imaging of c-Myc Transgenic Mice Identifies the Genotoxic N-Nitroso-Diethylamine as Carcinogen in a Short-Term Cancer Bioassay
Source: PLoS One. 2012 Feb 2;7(2):e30432. doi: 10.1371/journal.pone.0030432 (PMC3271108; doi:10.1371/journal.pone.0030432)

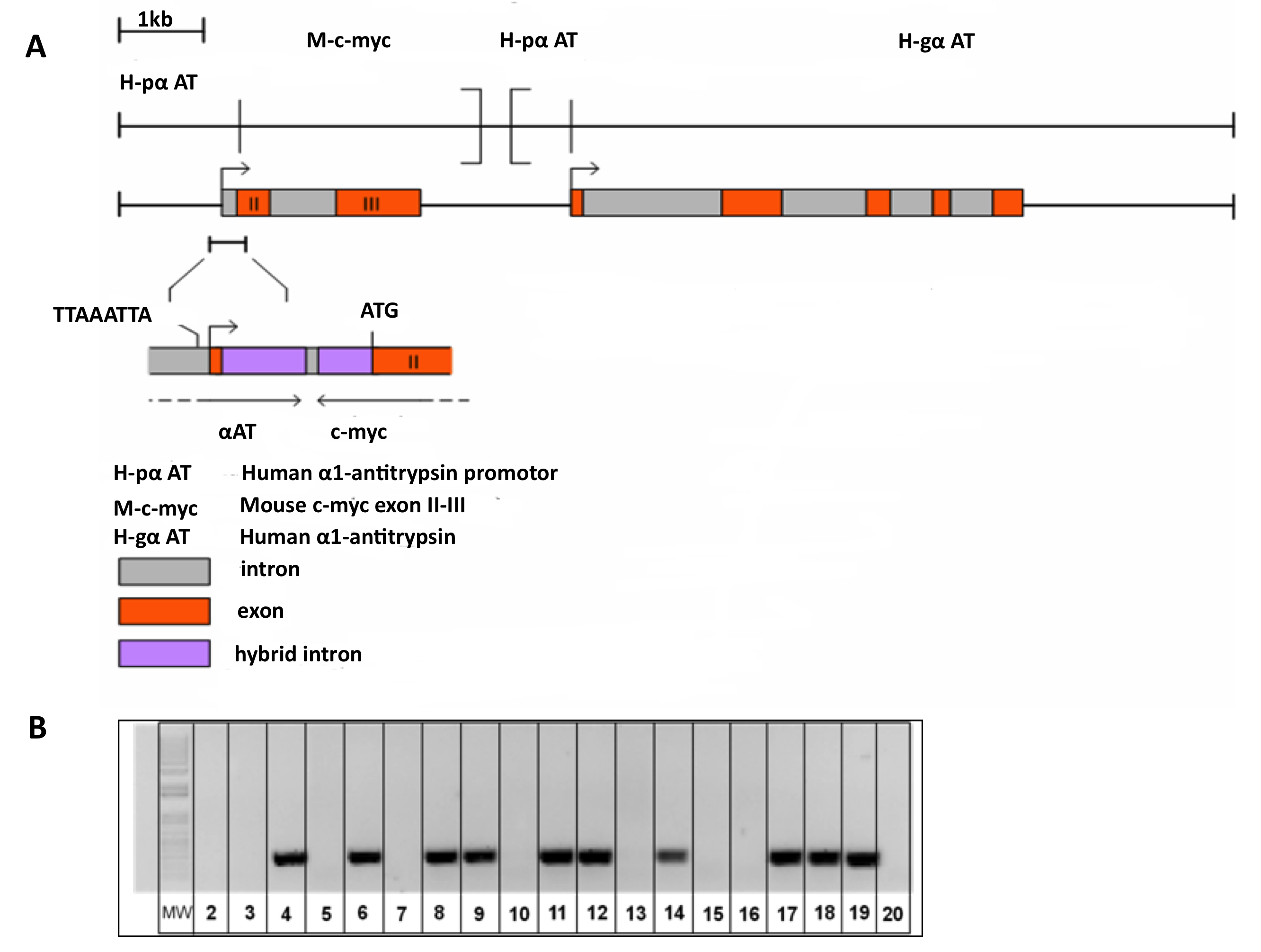

Supplement: Figure S1 — Scheme of the gene construct for the production of c-Myc transgenic mice. (A) The gene construct contains the sequence of the α1-antitrypsine promoter thereby enabling liver specific gene expression of c-Myc. Based on ongoing research evidence was obtained that this genetic model of liver cancer undergoes different stages of disease with initial low and high grade dysplasia followed by different grades of hepatocellular carcinoma as evidenced by histopathology. (B) Depicted is an ethidium bromide stained agarose gel of a 303 bp PCR amplification product of the transgene detected in individual animals; the lanes 2, 3, 7, 10, 13, 15, and 16 refer to non-transgenic animals (no band) while the lanes 4, 6, 8, 9, 11, 12, 14, 17, 18 and 19 are transgenic animals. Note, lane 20 is a mock (water) negative control. MW = molecular weight marker. (TIF) [file pone.0030432.s001.tif]

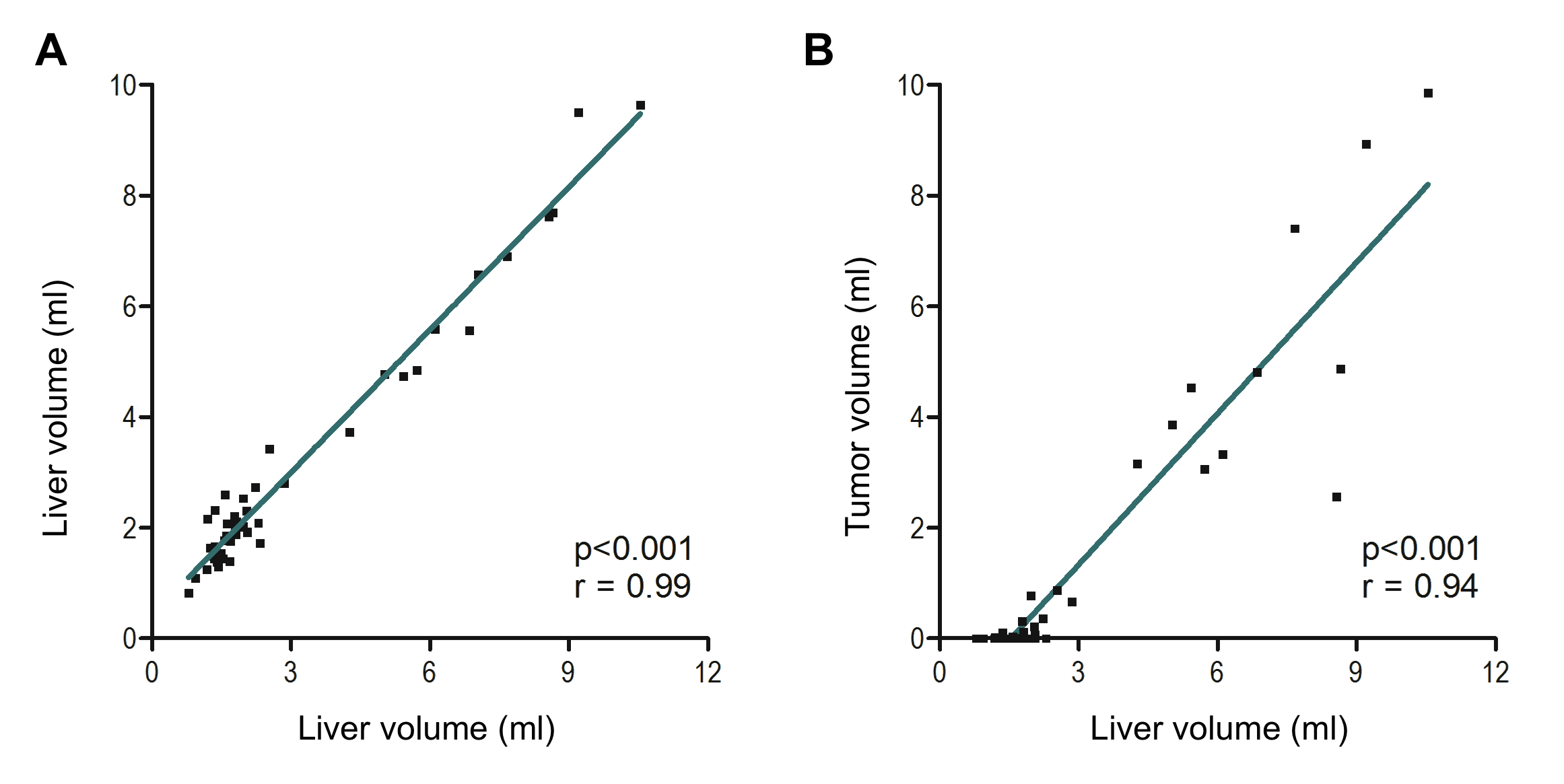

Supplement: Figure S2 — Correlation between liver weight, liver volume and tumor volume of c-Myc transgenic mice. (A) demonstrates the close correlation between the liver weight and the liver volume (p<0.001, r = 0.99). (B) The correlation between liver volume and tumor volume – as determined by CT – is shown (p<0.001, r = 0.94). (TIF) [file pone.0030432.s002.tif]

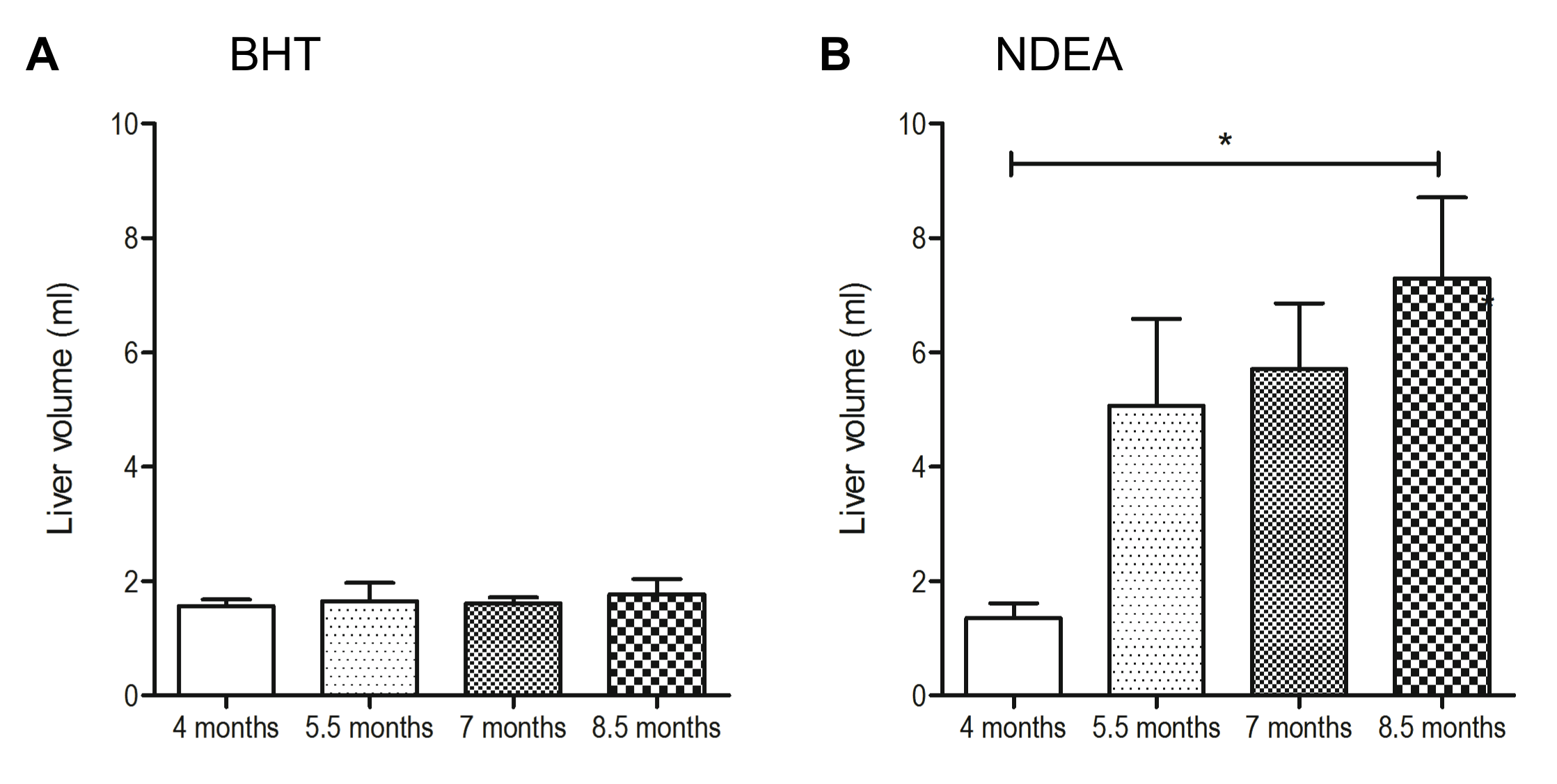

Supplement: Figure S3 — Liver volume in BHT- and in NDEA-treated c-Myc transgenic mice at different stages of disease. Liver volume as determined by semiautomatic segmentation in contrast-enhanced computed tomography was constant over time in BHT-treated animals (A), whereas in NDE-treated animals it increased till the age of 8.5 months (B). (TIF) [file pone.0030432.s003.tif]
